# Trustworthiness of the electronic health record in Germany: an exploratory, user-centered analysis

**DOI:** 10.3389/fdgth.2025.1473326

**Published:** 2025-03-07

**Authors:** Niklas von Kalckreuth, Marvin Kopka, Christine Schmid, Cornelia Kratzer, Anna Reptuschenko, Markus A. Feufel

**Affiliations:** Division of Ergonomics, Department of Psychology and Ergonomics (IPA), Technische Universität Berlin, Berlin, Germany

**Keywords:** electronic health record, eHealth, trustworthiness, privacy, data security

## Abstract

**Introduction:**

The integration of Electronic Health Records (EHRs) offers significant potential to improve patient care and reduce costs. In Germany, every patient will be provided with an EHR starting January 2025. However, the success of EHRs depends on patient trust and usage. Understanding the factors that shape perceived trustworthiness is crucial, yet EHR-specific research remains limited.

**Methods:**

To explore key factors influencing initial trust, 30 German participants interacted with a functionally realistic EHR prototype. Semi-structured interviews were conducted to evaluate its trustworthiness and suggest improvements.

**Results:**

Thematic analysis identified five key themes: Provider Reputation, User Feedback, User Experience of Contents, User Experience of Functions, and User Data Control.

**Discussion:**

Fostering trust in the German EHR requires attention to technical features and contextual factors. Beyond provider reputation, three aspects emerged as central: (a) professional visual and user experience design to enhance usability and signal reliability; (b) accurate, clear content to foster transparency; and (c) user empowerment through intuitive data control and accessible support. Transparent communication about GDPR compliance further strengthens trust and supports adoption. In Germany's tightly regulated environment, clearly conveying EHR security standards, providing robust support, and leveraging social proof can significantly enhance trust and drive adoption of digital health solutions.

## 1 Introduction

Digital transformation is playing an increasingly important role in healthcare (1–3). An essential aspect of digital transformation in healthcare is improving connectivity between actors in the healthcare system (2, 4), which is facilitated by the transition from paper-based patient records to Electronic Health Records (EHRs) (2, 3). Although EHRs are already in use in various countries, their adoption, functions, and implementation timelines differ internationally. In Germany, the EHR was rolled out nationwide on January 15, 2025, and was provided to every patient by default unless they actively opt out (5), offering the combined functions of an EHR and a personal patient record. The system is provided by health insurance companies, whereas patients, physicians, and hospitals provide the data to be uploaded and stored in the EHR. A unique feature of the German EHR is that patients have full access to and control over their data through mobile apps provided by their health insurance providers (6, 7). They can upload their own data and delete all stored data (including those uploaded by physicians and hospitals) at any time. Hence, data sovereignty rests solely with the patients (3, 8). The EHR offers numerous opportunities to improve patient care, as digital communication between different institutions can make care more effective, efficient, and safer (9, 10). However, concerns about data protection and security lead many people to avoid using EHRs, a behavior observed in multiple countries, including Germany, France, Australia, and the United Kingdom (3, 9, 11–14). To mitigate the perceived risks and foster willingness to share health data via the EHR, trust in both the security of stored personal health data within the EHR system and the healthcare providers managing them has been shown to be an essential factor (9, 11, 15, 16).

Trust is a psychological state that involves the intention to accept vulnerability based on positive expectations of another's intentions or behavior (17). A trust relationship requires at least two parties: the trustor (e.g., the technology user) and a trustee (e.g., the technology and its provider) (18). Trust inherently involves vulnerability and risk (19, 20), such as the potential insufficient protection or misuse of sensitive health data in the context of EHRs (11, 21–23). Trust serves the purpose of reducing complexity, e.g., by supporting the trustor in actions or decisions (24, 25). In human-technology interactions, trust formation depends on prior user experience. Whereas existing experiences foster knowledge-based trust, *initial trust* relies on user perceptions when prior experience is absent (26, 27). As the use of EHRs is not yet widespread in Germany due to factors such as strict data protection regulations, limited IT infrastructure in some healthcare settings, and a lack of awareness among patients and providers (10, 28), and users have concerns about their initial use, we focus on initial trust in this paper.

Various domain-general trust models highlight that initial trust is shaped by both individual tendencies to trust (a personality trait reflecting one's general willingness to trust) and the perceived trustworthiness of the trustee (specific cues that signal trustworthiness (19, 24, 26, 29, 30). Previous research has identified several factors influencing users' perceived trustworthiness in digital contexts (18, 30, 31). In eCommerce and eGovernance systems, these factors included user experience, data protection, digital security features and the developer's reputation (18, 30). In contrast, perceived trustworthiness in eHealth applications depends more on the users' data autonomy, the quality of the content, and the comprehensibility and reliability of the system (31). Regardless of the domain, the visual design of a technology strongly influenced trustworthiness (18, 30, 31). Overall, these findings suggest that the antecedents of trustworthiness and their relative importance vary depending on the type of system (e.g., apps or websites) and the domain (19, 29). The technology- and domain-specific factors influencing trust formation during the interaction with the EHR remain thus far under-researched. In this study, we explore the factors influencing the perceived trustworthiness of the EHR among users in Germany.

Given the lack of user research and to provide a detailed description of EHR users' perceptions and experiences and how they influence trust in EHR systems, we chose an exploratory approach in which we conducted semi-structured interviews following a sample interaction with a functionally realistic, interactive EHR prototype (click dummy). The following section details our study methodology.

## 2 Methods

### 2.1 Participants

The interviews were part of a larger mixed-methods study that investigated privacy perception and interaction with EHRs, specifically how different characteristics of health data influence users' willingness to upload medical findings (32). The study was conducted from March 1, 2022, to May 15, 2022. We aimed for a total sample size of 30 participants, following the recommendations for sample extensiveness for individual interviews (33). Recruitment continued until data saturation was reached, meaning that no new insights emerged in subsequent interviews. Individuals aged 18 years and older residing in Germany were allowed to participate in the study to reflect the intended user group of the EHR in Germany. Participants were insured under the statutory health insurance system in Germany. The investigators invited graduate students to participate via our university's study participation portal (SONA), which is used to recruit participants for research studies. Students enrolled in the Human Factors master's program can register on the platform, as they are required to complete participation hours as part of their curriculum. Participants could voluntarily sign up for available time slots. Before the beginning of the study, participants gave written informed consent according to the ethical guidelines of the German Research Foundation. A total of 30 participants (13 male, 17 female) were interviewed. The interviews were conducted by trained research assistants with a background in Human Factors and qualitative research methods. To minimize interviewer bias, a neutral stance was maintained throughout the interviews, and predefined open-ended questions were used. Furthermore, the interviewers were not insured with BARMER, ensuring that they did not have pre-existing familiarity or bias toward the specific EHR application used as the prototype. The interviews lasted between 18:31 min and 27:13 min, with an average duration of 22:44 min. Table 1 describes the demographic characteristics of the sample in detail.

**Table 1:** Sample demographics.

Demographic characteristic	Respondents
Age (years), mean (SD)	31.67, (9.86)
Gender, *n* (%)	
Male	13 (43.3)
Female	17 (56.7)
Education, *n* (%)	
General qualification for university entrance	2 (6.7)
University degree (bachelor or master)	25 (83.3)
Ph.D.	1 (3.3)
Other	3 (10)
Experience with mHealth applications, *n* (%)	
Regular use of mHealth applications	21 (70)
No use of mHealth applications	9 (30)

### 2.2 Materials

**Figure 1:**
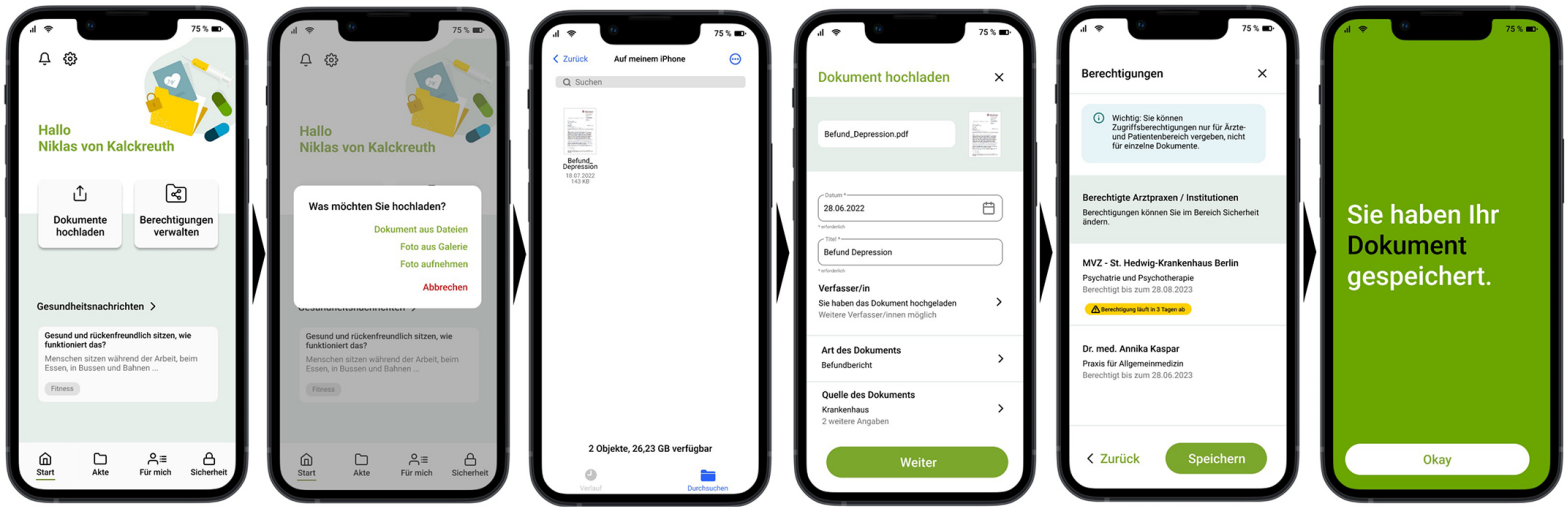
Example click sequence for uploading medical data to the EHR prototype.

To allow for a realistic interaction with an EHR, a software for interface design (named Figma) was used to create a realistic interactive prototype (i.e., a click dummy) based on the model of the mobile EHR application of a German health insurance (BARMER)—the eCare app. Specifically, the prototype allowed participants to upload findings (see Figure 1), grant or revoke permissions to view findings, and create medication plans. We self-constructed the interview guideline (see Supplementary Material) following standards for constructing interview questions, including the use of simple language, short, specific, and neutral wording (e.g., no leading questions or double negation) and one-dimensionality, that is, each question could only refer to a single fact (34, 35). The interview guide consisted of a total of five main questions and eight subquestions in the main section, as well as one introductory and one closing question. The introduction included two additional subquestions.

### 2.3 Procedure

The study entailed semi-structured interviews with participants following interaction with the EHR app. During the interaction part, the participants completed tasks designed to familiarize themselves with the core functionalities of the EHR app. These included reviewing data protection and security information, uploading a medical document, storing emergency contact and health information, managing access permissions for healthcare providers, and organizing a medication plan. The semi-structured interview were then conducted by a study assistant. Participants were asked to evaluate their interaction with the EHR app, give their general assessment of its trustworthiness, and indicate which features/information they think should be improved or added to increase the trustworthiness of the EHR. At the end of the study, demographic data of the participants were collected using a questionnaire.

### 2.4 Analysis

The interviews were verbatim transcribed by two study assistants using the *f4tranSkript* software. The transcription was complete with selective utterance and logging of paraverbal and non-verbal elements. Responses were analyzed using the inductive thematic analysis approach described by Braun and Clarke (36) and coded using the MAXQDA2022 software. First, all interview transcripts were carefully read to identify meaningful units of text related to the perceived trustworthiness of EHRs. In our coding process, we systematically coded entire sentences rather than paraphrased segments to maintain the original meaning and context of participants' statements. Second, units of text addressing similar aspects were grouped into analytic categories, with provisional definitions assigned to each. A single unit of text could be included in multiple categories where applicable. Third, the categories were reviewed to ensure clear definitions, consistency, and representative supporting data. To ensure reliability, codes were reviewed and discussed among the research team until consensus was reached. Following the inductive thematic analysis approach, we first identified 40 codes from the coded data. These codes were then grouped into 13 subthemes, which were further clustered into 5 overarching themes, representing key factors influencing trustworthiness perceptions. A detailed overview of the coding structure, including all main themes, subthemes, individual codes, the number of participants per code, and illustrative examples, is provided in the Supplementary Materials.

## 3 Results

Thematic analysis of the interviews resulted in five themes that influence the perceived trustworthiness of EHR apps according to the participants namely: provider reputation, user feedback, user experience of contents, user experience of functions, user data control.

**Figure 2:**
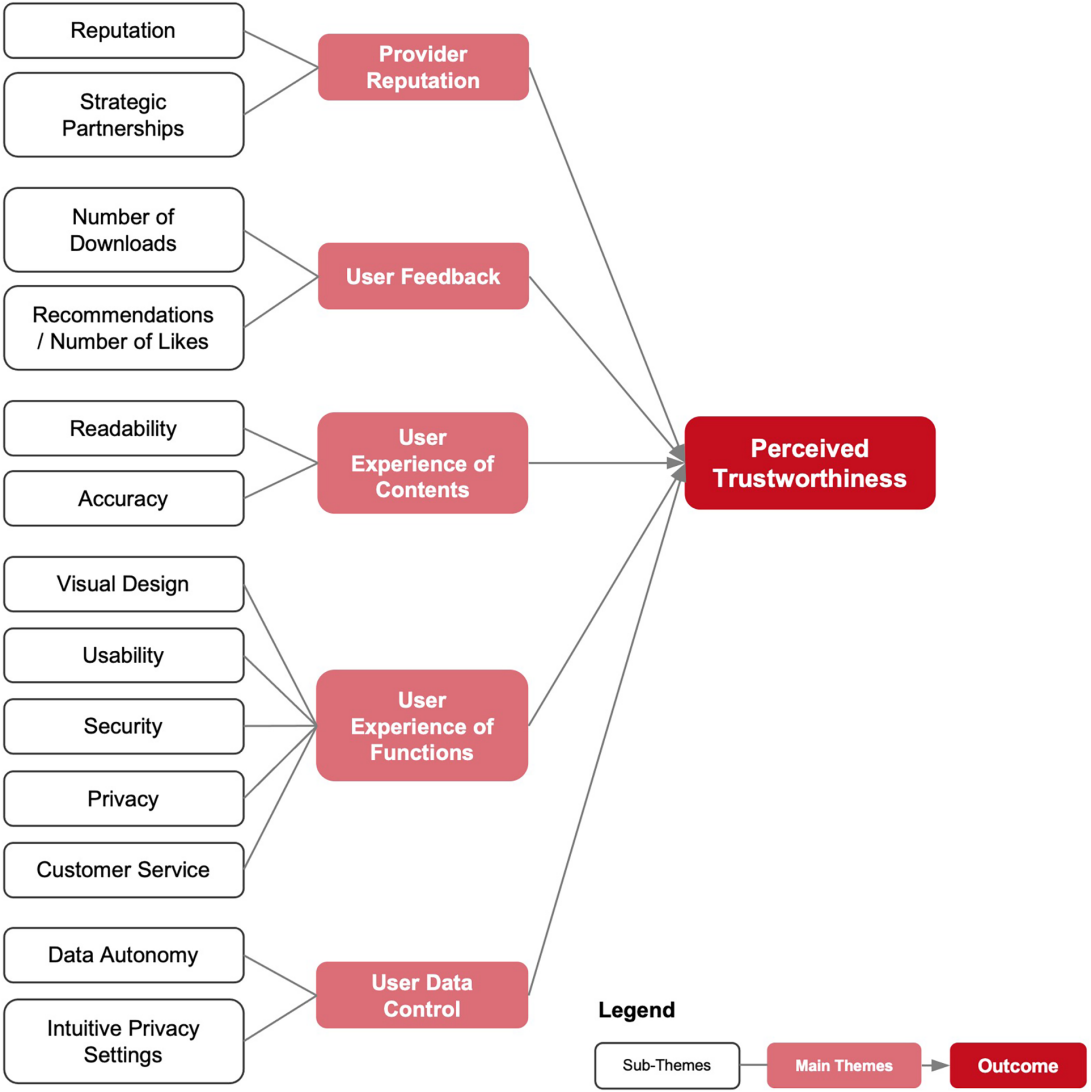
Thematic map of the analysis—dark red boxes represent the outcome variable, light red boxes indicate main themes, and white boxes depict sub-themes.

In the following section, these five major themes and their respective sub-themes are presented in more detail. Quotes have been provided to illustrate facets of each theme. Figure 2 presents all themes and sub-themes in the form of a thematic map.

### 3.1 Provider reputation

#### 3.1.1 Reputation

Most participants associated digital products from well-known providers with higher trustworthiness compared to those from unknown or negatively perceived providers. A significant factor for establishing a reputation is, for instance, prior interaction with the company, e.g., through another app of the provider that is already in use, which increases the level of awareness of the provider and decreases the need to verify expectations about product quality.

[…] because I am already familiar with this from other apps from the provider and have already built-up trust with it. (P#01)

Additionally, for various participants, the provider's public image played a decisive factor in trust formation, particularly regarding data privacy standards and certifications by independent institutions.

If it was a company that I didn't know beforehand or an app that was recommended to me by friends or acquaintances, then I would find out more. With an institution like [name of provider], where I've been a customer virtually my whole life, I assume that they work properly. And they already have my trust in advance, so to speak […]. (P#10)

If there was a scandal involving data theft at a healthcare provider and I heard about it in the media, I certainly wouldn't want to use the app. (P#28)

#### 3.1.2 Strategic partnerships

The trustworthiness of the EHR also depends on the reputation of a provider's *strategic partnerships*. for instance, when a provider without technological expertise, such as a health insurance company, teams up with a reputable technology firm. Some participants emphasized the importance of knowing which companies are involved in the development and operation of the system.

And it would be good to know which other companies are involved to make things a bit clearer. (P#24)

Moreover, numerous participants stated, that the level of trustworthiness of an EHR depends on the companies that are tasked with developing the digital health app and managing the data. The involvement of a well-known and respected technology firm as a partner markedly boosts the perceived trustworthiness of the provider.

So, from what I have read in the privacy policy where the cooperation partner is mentioned, that the data is stored by IBM Germany on a German server, I assume that I can trust that very much. (P#10)

### 3.2 User feedback

#### 3.2.1 Number of downloads

Several participants mentioned high download numbers in the app store as a source of trust in the app. This user feedback was interpreted as proof that the app has no negative consequences, and that the technology is mature.

Yes, of course, the more people use it, and nothing happens, nothing bad happens somehow with the data, it was not tapped. Then, naturally, this enhances the trustworthiness, and consequently, it boosts its adoption. (P#23)

#### 3.2.2 Recommendations/number of likes

Recommendations have a positive influence on the perceived trustworthiness of the EHR app. Some participants highlighted that recommendations from their personal environment, including family, friends, and acquaintances, played a crucial role in shaping trust. If someone in their social environment used the application and recommended it, they perceived a lower risk of negative consequences.

[I follow] personal recommendations from friends or families, if they say the website is okay, it is quite trustworthy for something like that, then I do it. (P#16)

In addition, many participants emphasized the role of user-driven feedback, such as likes, ratings, and positive comments. They viewed product reviews as particularly impactful, as they considered other users to be expert evaluators whose experiences reinforced the app's trustworthiness.

Yes, so of course you first look for trustworthy sources, for example reviews or ratings and comments in the App Store to see how it is rated by others. (P#09)

### 3.3 User experience of contents

#### 3.3.1 Readability

The perceived trustworthiness of EHR is increased when information is easy to comprehend. Many of the participants emphasized the importance of well-structured texts that clearly highlight critical and essential information, perceiving them as more transparent and trustworthy.

Okay, the text was really well-structured and relatively easy to access. That way, I could skim through it much faster and understand what it was actually about. It's important to me that something like this is implemented in an app. (P#10)

Additionally, diverse participants pointed out that language complexity affects trust perceptions. Overly complex texts, characterized by technical jargon and challenging readability, were seen as problematic, as they created the impression that vital information was being concealed or intentionally made intransparent.

And in fact, this information aspect is always there, so when I want to find out what exactly is happening, I have easy access to the information and can find what I'm looking for quite transparently. That this is not hidden in such endless sentences or in any complex formulations, but that it is explained in a clear and simple way that is easy to understand. […] For me, this is the key factor that shows that the provider is trustworthy. (P#15)

#### 3.3.2 Accuracy

The accuracy of information plays a significant role in shaping perceived trustworthiness of EHR and encompasses two key elements. First, accuracy relates to the *consistency of the information* provided via the technology. Some participants emphasized the importance of semantic coherence, ensuring that information across different sections of the app remains consistent and free from contradictions.

Yes, if things don't match, for example. For example, if information on the same topic doesn't match in different places in the app or something like that, then I wouldn't do it anymore, I would cancel it. (P#19)

Similarly, a few participants highlighted the need for syntactic consistency, meaning that word choice and sentence structure should be uniform throughout all texts.

For me, it's important that the language remains consistent. It would be strange if certain passages had a completely different tone. (P#01)

Second, correct spelling was mentioned by various participants as a crucial factor. Typographical errors in the app's text were perceived as a sign of unprofessionalism and raised concerns about the quality of technical aspects such as data security, too. Overall, inaccurate and/or inconsistent information was as seen as diminishing trust in the EHR.

I can't describe it exactly, but I would say that it's kind of, so if the app also had many typing errors, then I wouldn't feel safe either, although it has nothing to do with data security. (P#21)

### 3.4 User experience of functions

#### 3.4.1 Visual design

The visual and aesthetic design of an EHR app influences its perceived trustworthiness. Numerous participants emphasized the importance of an attractive design, noting that visually appealing layouts contribute to a more positive perception of the app.

Yes, it seems trustworthy to me. The design was nice, and it didn't look too complicated. (P#20)

Also, the majority of the participants associated a professional design with careful attention to detail, linking it to compliance with data security and privacy requirements.

If the app is well-designed and looks professionally made, I'd say it also increases trustworthiness. In contrast, if it looks like it's from the ‘90s, it's a different story. (P#06)

Some participants specifically highlighted modernity as a key factor, associating a contemporary design with regular updates that ensure the app remains up to date.

Because it looks modern, and it also gives the impression that it is regularly updated, maintained, and adjusted to current guidelines and regulations. If something looks like it's ten years old, you naturally start wondering whether it still meets modern standards. (P#07)

Furthermore, Several participants noted that familiarity with design elements from other apps fostered trust, as known and valued features created a sense of reliability. In summary, a professional visual design is regarded as a standard for high-quality apps, and any deviations are promptly interpreted as questionable and untrustworthy.

So even if the design seems familiar to me and I think to myself, ah yes, that's how it was presented graphically in another app, for example, that you had to upload a document, then I'm more likely to trust it than if a colorful window with lots of different texts suddenly appeared or something like that. (P#29)

#### 3.4.2 Usability

Usability––that is the ease and efficiency with which users can achieve specific goals within a particular context––significantly influences the perceived trustworthiness of an EHR. Most of the participants emphasized the importance of system consistency, noting that bugs, formatting errors, or broken links were interpreted as signs of negligence on the part of the provider.

An app seems trustworthy to me, of course, if it works properly. If there are too many bugs, I wouldn't trust it. (P#04)

So, I always get suspicious when I use an app and I notice that it doesn't work properly or some buttons don't actually work or it takes a really long time to load or some things move strangely, then I get suspicious. And if the app were to be optimized and improved, then the trustworthiness would definitely increase even further. (P#01)

Additionally, a few participants highlighted the role of efficiency, stating that well-designed processes appeared trustworthy, while redundancies in searching or retrieving data were met with skepticism. These deficiencies in system consistency and efficiency were often projected onto broader concerns about data security, ultimately diminishing the EHR's overall trustworthiness.

Exactly, within a single process, without too many steps in between or an excessive number of tabs opening. A clear and structured flow always provides a sense of security. (P#14)

Expectation conformity was another critical factor for some participants, who noted that trust was influenced by the extent to which the system's processes aligned with their expectations.

If the app is comprehensible for the user. Meaning it's clear which categories it operates in and, most importantly, how one can manage their data. You have a certain expectation, and if that expectation is met, that's definitely a good thing. (P#03)

Finally, ease of use was the most frequently mentioned aspect, with the majority of all participants emphasizing that intuitive navigation and straightforward workflows were essential for fostering trust in the EHR.

That I get the feeling they actually want me to find my way around quickly. There are often apps where you first need to complete a special course just to understand how everything works. But that wasn't the case here. You could figure out where to go pretty quickly. (P#10)

#### 3.4.3 Security

The security of an EHR is defined by two elements: data security and access security. Data security concentrates on the protection and integrity of the data collected in the app, emphasizing the need for a convincing security framework. Many participants highlighted the importance of secure data transmission, stressing that encryption and other protective measures must be in place to prevent unauthorized access.

I basically only pay attention to whether the data is transferred encrypted or unencrypted. If it's encrypted, I trust that everything is fine during the data transfer. (P#25)

Also, several participants emphasized the need for secure data storage to ensure sensitive health information remains protected at all times.

It's important to me that the data is reliably stored and that if I save it in the app, I can always find it again. That means it also needs to be securely backed up on the backend so that technical errors don't suddenly cause my data to disappear. And the data should also be stored in an encrypted manner so that it can't be easily hacked. (P#30)

Most of the participants underscored the importance of limiting data access strictly to authorized entities, while a handful of participants noted that masking personal data within the app could further enhance security.

The most important thing, especially when it comes to sensitive data like medical data, is ensuring that within the institution, only those who are directly involved have access. This means that not just anyone working there should have access to the data by default—they should need a specific reason to access it. (P#25)

That when I click on the ‘Personal Data' section, everything is encrypted except maybe my name—so that no sensitive details, like a password, are displayed in plain text. (P#14)

Access security, conversely, focuses on preventing third-party access to the app, especially if a mobile device is being used by someone other than the owner, e.g., to take a quick photo. Many participants stressed the importance of strong authentication methods, such as two-factor authentication using One-Time Passwords (OTP) or Transaction Authentication Numbers (TAN) in combination with passwords or biometric verification.

But that I can only get into the app with a password and a TAN or OTP, for example, and not any strangers who find my cell phone if I lose it, for example. That they can't access it. That would be very trustworthy for me. (P#24)

Furthermore, numerous participants emphasized that authentication should not only occur at login but also be required for subsequent interactions involving sensitive data.

In retrospect, it might actually be even more important to confirm at some point in between that the person accessing all the sensitive data is really the one who is logged in. (P#09)

These components—data security and access security—are regarded by users as fundamental to the app's trustworthiness, with any shortcomings in authentication quickly perceived as security flaws, consequently labeling the application as untrustworthy.

#### 3.4.4 Privacy

In the context of the EHR, data privacy focuses on data protection, including the storage, processing, and sharing of only the data that is necessary to serve the user's interest. Many participants emphasized that effective data protection measures must be in place to safeguard sensitive health data from unauthorized access.

Um, I think the trustworthiness is actually higher when you can see that the developers—or, yeah, exactly, that they have engaged with data protection and are also complying with relevant laws. I believe that's an important point. (P#23)

Additionally, diverse participants highlighted the importance of data minimization, stressing that only the information strictly required for healthcare purposes should be collected and processed.

Uh, exactly. And that only data is stored for a comprehensible reason. For example, it's clear why your name is stored—otherwise, the app wouldn't really work. But for other things that might not necessarily need to be stored, they simply shouldn't be stored. (P#04)

Transparency in data processing was a major concern, with a great number of participants stating that clear disclosure and justification of data storage, processing, and sharing practices are essential for fostering trust. Users want to understand what happens to their data at each stage of its lifecycle.

I think trust also comes more from the fact that there are plenty of notices explaining things like ‘How is my data processed? Is it shared with others?'—so that you can ultimately view all of this information. (P#29)

Furthermore, many participants specifically mentioned that the geographical location of servers significantly impacts their trust in the system. They expressed a strong preference for servers located in Europe—Germany, in particular—due to the rigorous data protection standards enforced by regulations such as the General Data Protection Regulation (GDPR). Users are particularly sensitive to gaps in data privacy practices, where deficits in any of the mentioned aspects can significantly undermine the perceived trustworthiness of the EHR.

So, from what I've read in the privacy policy, for example, that the data is stored on German servers. In other words, that the data protection laws of Germany really do apply to it. I assume that I can trust that very much. (P#30)

#### 3.4.5 Customer service

Some participants found it trustworthy if they can contact the EHR provider quickly and easily. They thus searched for a contact button or an option to personally contact the company (e.g., via phone).

I don't know now. If there would have always been a prominent help or contact button somewhere where you could contact someone directly, I still find something like that trustworthy and helpful if you know you can reach someone by phone. (P#29)

Diverse participants emphasized that direct personal contact was favored, as chatbots or electronic announcements were not seen as an adequate alternative in this case.

[…] and also contact options, such as a service hotline or an email function, so that there is always a direct point of contact—not through a chatbot or anything like that—in case any problems arise. (P#01)

### 3.5 User data control

#### 3.5.1 Data autonomy

Data autonomy refers to user control over the collection, storage, processing, and sharing of their data. If data autonomy is perceived by the participants, it increases the trustworthiness of the EHR app. The ability to control how data is processed was particularly relevant, as emphasized by nearly all the participants.

Um, yeah, I think there are actually some settings where you can choose which medical practices should have access to which of your data. I saw that as a positive aspect. (P#23)

Additionally, many participants highlighted the importance of being able to manage data sharing preferences. The presence of a comprehensive data inventory, ensuring transparency about all stored, processed, or shared information, was considered essential by four participants.

That would of course be very trustworthy, as I said earlier, if you had a complete overview of, okay, what I have uploaded, what data has been saved and what has been shared with other parties. (P#11)

Likewise, some participants stressed the need for an easy way to delete data, reinforcing the desire for full control over personal information in an EHR app.

Yes. I can basically always view everything and, as far as I've seen, I could also delete something and remove it again in the end. I had a good impression—it seemed trustworthy. (P#14)

#### 3.5.2 Intuitive privacy settings

Respondents emphasized the importance of being able to configure privacy settings according to their preferences, with many participants reporting reduced trustworthiness if privacy settings could not be easily accessed or adjusted.

If it's an app that doesn't give me the feeling at all that I can easily change settings somewhere, e.g., that the data are anonymized, then I would think three times about uploading something. (P#04)

Various participants specifically noted that privacy settings should be readily accessible in the menu and ideally presented prominently during the initial use of the app. If adjusting settings was perceived as overly complicated, it raised doubts about the overall utility and trustworthiness of the app.

If it's an app that doesn't give me the feeling at all that I can easily change settings somewhere, e.g., that the data are anonymized, then I would think three times about uploading something. (P#04)

In addition, some participants suggested the implementation of predefined data usage profiles (e.g., from “extremely private” to “very public”) to simplify privacy management, as they preferred streamlined choices over intricate details to exert control.

In other words, I think it's good if there are certain data usage profiles, for example, so that you can relatively easily choose the profile, the data protection profile that best suits your own preferences, such as from “extremely private” to “very public”. (P#27)

## 4 Discussion

The results show that the perceived trustworthiness of EHR apps is shaped not only by technical features such as design, usability, and privacy but also by broader context factors, particularly the provider's reputation and public image. Our findings extend prior research by demonstrating that this reputation is not solely tied to the provider itself but also shaped by the strategic partnerships formed during the system's development. In the case of the German EHR, health insurance companies collaborate with well-established IT firms, and this partnership itself serves as a credibility signal. This expands upon previous studies where reputation was primarily linked to the healthcare provider alone (18, 31). Our results suggest that users not only evaluate the provider's standing but also consider the perceived reliability and expertise of external technology partners involved in the EHR's implementation.

Additionally, our findings refine existing perspectives on user data control. While previous studies (9, 22, 31) address the general influence of data autonomy on the perceived trustworthiness of mHealth apps, our results offer a more nuanced perspective on what precisely data autonomy entails for users. Our participants underscored the critical need for a comprehensive data inventory and straightforward mechanisms to manage their health data, illustrating how such control directly bolsters the perceived trustworthiness of EHR systems. These findings show that responsible data handling by the provider plays a major role in the assessment of perceived trustworthiness and, consequently, the potential use of the EHR. This includes not only compliance with European data protection and security standards but also granting control over one's own health data and a simplified handling of privacy settings.

Unlike the trust dynamics observed in fitness and eCommerce apps where user trust hinges more on direct user experience and service quality (18, 31), our findings highlight that a distinct interplay of user perceptions and technical features shapes trustworthiness in the EHR domain in Germany. Some aspects such as using a professional design, avoiding bugs, orthographic errors, or other design deficits have already been shown to negatively influence trustworthiness of mHealth apps in previous studies (18, 30, 31). However, our results suggest that these deficits do not directly erode trustworthiness but rather impact it indirectly through their effect on perceived privacy and security. Participants associated such flaws with a lack of attention to potential risks to data protection and system reliability, which undermined their confidence in the system's ability to safeguard sensitive health data. This underscores how even minor design deficits can trigger broader concerns about data security and privacy, ultimately diminishing overall trustworthiness of a complex technology such as the EHR. Addressing these issues is key, as a professional and reliable user interface not only enhances usability but also reassures users of the system's commitment to responsible data handling.

Based on the results, there are several possibilities for EHR providers in Germany to increase the perceived trustworthiness of their applications and thus the likelihood of use. Prioritizing data privacy and security is crucial, which involves adhering to strict standards and enabling intuitive control over privacy settings. Additionally, it is essential to communicate about data protection and privacy transparently and understandably, for instance, by utilizing easy-to-understand privacy features (37, 38). This approach underscores the importance of making users feel informed and in control, thereby enhancing the trustworthiness of EHR applications. Moreover, the significant link between an EHR's visual design and usability and users' perceptions of data privacy and security highlights the importance of professional, error-free design in building trust. In other words, investments in visual appeal and user-friendliness not only foster trust but also demonstrate a commitment to safeguarding user data, offering a comprehensive strategy for enhancing EHR adoption in the German context.

### 4.1 Limitations and future directions

This study has several limitations that must be addressed in future research. Currently, very few individuals in Germany have personal experience with the EHR. We attempted to address this by using a functionally realistic EHR click dummy as the basis for the interview. Nevertheless, the study should be repeated after the nationwide introduction of the EHR in the entire healthcare system to validate the categories. Another limitation is selection bias, as most of the participants were young and highly educated. These are characteristics that influence the perception of privacy issues (39, 40). This first exploratory study should therefore be empirically validated with a representative sample and quantitative methods that allow drawing causal inferences, e.g., using conjoint analyses. Finally, the trustworthiness perception is highly context-dependent (19, 29). This study was tailored to the German EHR, which differs in many aspects from EHRs in other countries. In follow-up studies, factors influencing perceived trustworthiness of the EHR should be validated in other countries.

## 5 Conclusion

To foster trust in the German EHR and encourage their wider adoption, it is essential for stakeholders to focus on both technical features and contextual factors that influence the technology's perceived trustworthiness. While provider reputation and strategic IT partnerships are critical, our study emphasizes that trust depends on more than reputability. Specifically, three key aspects emerged as central to building trust. (a) A professional, modern design enhances usability and signals attention to detail, reinforcing users' confidence in the system's reliability and security. Design flaws or inconsistencies can undermine trust by triggering concerns about attention to detail and by extension data protection. (b) Consistency, clarity, and accuracy of information contents are paramount. Well-structured, easily understandable texts without technical jargon or spelling errors create a sense of transparency and professionalism, reducing perceived risks. (c) Empowering users through intuitive data management tools, privacy settings, and accessible customer support fosters a sense of control, which directly bolsters trust. Transparent communication about data handling and compliance with stringent data protection regulations, such as GDPR, is essential to improve users' trust perceptions and, ultimately, technology adoption.

Building initial trust is crucial, and it can be improved by offering reliable customer support, empowering users with control over their personal health data, and leveraging social reputation, for instance, based on high download rates and positive user ratings. By addressing these strategies, stakeholders can mitigate privacy concerns and enhance adoption rates, thereby unlocking the potential benefits of the EHR and, ultimately, contributing to the long-term success of the digital transformation in healthcare.

## Data Availability

The dataset analyzed in this study is available upon reasonable request. Requests to access these datasets should be directed to niklas.vkalckreuth@tu-berlin.de.
