# Supplementary material for: Trustworthiness of the electronic health record in Germany: an exploratory, user-centered analysis
Source: Front Digit Health. 2025 Mar 7;7:1473326. doi: 10.3389/fdgth.2025.1473326 (PMC11926143; doi:10.3389/fdgth.2025.1473326)
Supplement: Supplementary file 1 [file Datasheet1.pdf]

Supplementary Material

1 Interview Guide

Introduction:

0. By completing various tasks, you have explored the functionalities of the app. How would you rate the usability of the app?
   - How would you evaluate/describe the user interface of the app?
   - How do you perceive the app's usability?

Main Questions and Subquestions:

1. What did you find particularly positive or negative?
   - Do the mentioned features affect the trustworthiness of the app?

2. Can you name key features that influence your perception of trustworthiness?
   - What is important to you when handling your data?
   - What additional information should the app provide to ensure that you feel completely secure?
   - How would you evaluate the information on "data handling" and privacy in the example of eCARE?
   - Is the provided description of the information transparent and easy to understand?

3. What aspects do you particularly consider when uploading documents containing sensitive data?
   - Would you be willing to upload medical reports about severe conditions (e.g., sexually transmitted diseases, psychological therapy)? What factors influence your decision?

4. What impression does the ePA app give regarding your control over data and documents (permission management)?
   - Do you feel that you have full control over who can access your data?

5. How do you assess the trustworthiness of the app?
   - What information should be added to the app for the highest trustworthiness?

Closing Questions:

6. Would you recommend the ePA app to a friend? Please briefly explain your reasoning.
   - We have now reached the end of the interview. Is there anything you would like to add?

2 Code table

Main Theme	Sub-Theme	Code	Example
Provider Reputation	Reputation	Level of Awareness (Provider & Product)	*"Yes, from a well-known insurance company, where I actually expect them to consider and appreciate these things."* (P#10)
Provider Reputation	Reputation	Image	*"No, since it's a health insurance company, a highly respected institution, I have no concerns about that."* (P#25)
Provider Reputation	Strategic Partnerships	Involved Companies	*"And it would be good to know which other companies are involved to make things a bit clearer."* (P#24)
Provider Reputation	Strategic Partnerships	Well-known Cooperation Partner	*"So, from what I have read in the privacy policy where the cooperation partner is mentioned, that the data is stored by IBM Germany on a German server, I assume that I can trust that very much."* (P#10)
User Feedback	Number of Downloads	Number of Downloads in the App Store	*"Otherwise, I don't really pay that much attention to it, actually. I rather go with what seems familiar to me or when I see that other users have already downloaded it from the App Store."* (P#19)
User Feedback	Number of Downloads	Adoption Rate	*"So, what I, well, I can't really judge that now, but what would still be important in general is that it is widely used."* (P#7)
User Feedback	Recommendations / Number of Likes	User-driven feedback (likes / ratings)	*"Are there comments or reviews available, what do the overall ratings look like, and are there many likes?"* (P#09)
User Feedback	Recommendations / Number of Likes	Recommendations from friends and family	*"Yes, of course. The more people use it, and if you notice that all your family members and friends are using it without any issues, and they say, 'Hey, you should use it too,' then that naturally increases trustworthiness."* (P#07)
User Experience of Contents	Readability	Text Structure	*"Okay, the text was really well-structured and relatively easy to access. That way, I could skim through it much faster and understand what it was actually about. It's*

			important to me that something like this is implemented in an app." (P#10)
User Experience of Contents	Readability	Language Complexity	*"So that it's not hidden in overly long sentences or buried in some kind of legal jargon, but instead explained in a clear and easily understandable way. That definitely makes it much more trustworthy."* (P#30)
User Experience of Contents	Accuracy	Semantic Coherence	*"Yes, if things don't match, for example. For example, if information on the same topic doesn't match in different places in the app or something like that, then I wouldn't do it anymore, I would cancel it."* (P#19)
User Experience of Contents	Accuracy	Syntactic Consistency	*"For me, it's important that the language remains consistent. It would be strange if certain passages had a completely different tone"* (P#01)
User Experience of Contents	Accuracy	Correct Spelling	*"I can't describe it exactly, but I would say that it's kind of, so if the app also had many typing errors, then I wouldn't feel safe either, although it has nothing to do with data security."* (P#21)
User Experience of Functions	Visual Design	Attractiveness	*"Yes, it seems trustworthy to me. The design was nice, and it didn't look too complicated."* (P#20)
User Experience of Functions	Visual Design	Modern	*"Because it looks modern, and it also gives the impression that it is regularly updated, maintained, and adjusted to current guidelines and regulations. If something looks like it's ten years old, you naturally start wondering whether it still meets modern standards."* (P#07)
User Experience of Functions	Visual Design	Professional	*"If the app is well-designed and looks professionally made, I'd say it also increases trustworthiness. In contrast, if it looks like it's from the '90s, it's a different story."* (P#06)
User Experience of Functions	Visual Design	Design Familiarity	*"So even if the design seems familiar to me and I think to myself, ah yes, that's how it was presented graphically in another app, for example, that you had to upload a document, then I'm more likely to trust it than*

			if a colorful window with lots of different texts suddenly appeared or something like that." (P#29)
User Experience of Functions	Usability	System Consistency	*"An app seems trustworthy to me, of course, if it works properly. If there are too many bugs, I wouldn't trust it."* (P#04)
User Experience of Functions	Usability	Efficiency	*"Exactly, within a single process, without too many steps in between or an excessive number of tabs opening. A clear and structured flow always provides a sense of security."* (P#14)
User Experience of Functions	Usability	Expectation Conformity	*"If the app is comprehensible for the user. Meaning it's clear which categories it operates in and, most importantly, how one can manage their data. You have a certain expectation, and if that expectation is met, that's definitely a good thing."* (P#03)
User Experience of Functions	Usability	Ease of Use	*"That I get the feeling they actually want me to find my way around quickly. There are often apps where you first need to complete a special course just to understand how everything works. But that wasn't the case here. You could figure out where to go pretty quickly."* (P#10)
User Experience of Functions	Security	Secure Data Transfer	*"I basically only pay attention to whether the data is transferred encrypted or unencrypted. If it's encrypted, I trust that everything is fine during the data transfer."* (P#25)
User Experience of Functions	Security	Secure Data Storage	*"It's important to me that the data is reliably stored and that if I save it in the app, I can always find it again. That means it also needs to be securely backed up on the backend so that technical errors don't suddenly cause my data to disappear. And the data should also be stored in an encrypted manner so that it can't be easily hacked."* (P#30)
User Experience of Functions	Security	Limited Data Access	*"The most important thing, especially when it comes to sensitive data like medical data, is ensuring that within the institution, only those who are directly involved have access.*

			This means that not just anyone working there should have access to the data by default—they should need a specific reason to access it.” (P#25)
User Experience of Functions	Security	Masking Personal Data	*“That when I click on the 'Personal Data' section, everything is encrypted except maybe my name—so that no sensitive details, like a password, are displayed in plain text.”* (P#14)
User Experience of Functions	Security	Authentication Method	*“But that I can only get into the app with a password and a TAN or OTP, for example, and not any strangers who find my cell phone if I lose it, for example. That they can't access it. That would be very trustworthy for me.”* (P#24)
User Experience of Functions	Security	Authentication Timing	*"In retrospect, it might actually be even more important to confirm at some point in between that the person accessing all the sensitive data is really the one who is logged in."* (P#09)
User Experience of Functions	Privacy	Data Protection Measures	*"Um, I think the trustworthiness is actually higher when you can see that the developers—or, yeah, exactly, that they have engaged with data protection and are also complying with relevant laws. I believe that’s an important point."* (P#23)
User Experience of Functions	Privacy	Data Minimization	*"Uh, exactly. And that only data is stored for a comprehensible reason. For example, it’s clear why your name is stored—otherwise, the app wouldn’t really work. But for other things that might not necessarily need to be stored, they simply shouldn’t be stored."* (P#04)
User Experience of Functions	Privacy	Transparency in Data Processing	*"I think trust also comes more from the fact that there are plenty of notices explaining things like 'How is my data processed? Is it shared with others?'—so that you can ultimately view all of this information."* (P#29)

User Experience of Functions	Privacy	Server Location & Jurisdiction	*"It would definitely be important to me that the data is stored only in Germany, so that Germany's data protection regulations fully apply to it."* (P#30)
User Experience of Functions	Customer Service	Personal Contact	*"[...] and also contact options, such as a service hotline or an email function, so that there is always a direct point of contact—not through a chatbot or anything like that—in case any problems arise."* (P#01)
User Experience of Functions	Customer Service	Easy Contact Option	*"I don't know now. If there would have always been a prominent help or contact button somewhere where you could contact someone directly, I still find something like that trustworthy and helpful if you know you can reach someone by phone."* (P#29)
User Data Control	Data Autonomy	Control over Data Sharing	*"Um, yeah, I think there are actually some settings where you can choose which medical practices should have access to which of your data. I saw that as a positive aspect."* (P#23)
User Data Control	Data Autonomy	Control over Data Processing	*"Mhh. Yeah, that you can control that they are not shared or processed further. And I think, especially when it comes to health apps, we're talking about really personal data."* (P#06)
User Data Control	Data Autonomy	Data Deletion	*"Yes. I can basically always view everything and, as far as I've seen, I could also delete something and remove it again in the end. I had a good impression—it seemed trustworthy."* (P#14)
User Data Control	Data Autonomy	Data Inventory	*"That would of course be very trustworthy, as I said earlier, if you had a complete overview of, okay, what I have uploaded, what data has been saved and what has been shared with other parties."* (P#11)
User Data Control	Intuitive Privacy Settings	Timing of Privacy Settings	*"Yeah, and what I personally think—though of course, this is also a question of preference—is that, especially when dealing with sensitive data, it would be nice if you*

			could set all privacy settings right away the first time you open the app." (P#19)
User Data Control	Intuitive Privacy Settings	Ease of Privacy Settings	*"If it's an app that doesn't give me the feeling at all that I can easily change settings somewhere, e.g., that the data are anonymized, then I would think three times about uploading something."* (P#04)
User Data Control	Intuitive Privacy Settings	Data Usage Profiles	*"That means I think it's good when there are transparent labels for certain data usage profiles, so you can easily choose the privacy profile that best fits your preferences—for example, from 'highly private' to 'very public.'"* (P#27)
